# Supplementary material for: Comprehensive Analyses of the Histone Deacetylases Tuin (HDT) Gene Family in Brassicaceae Reveals Their Roles in Stress Response
Source: Int J Mol Sci. 2022 Dec 28;24(1):525. doi: 10.3390/ijms24010525 (PMC9820156; doi:10.3390/ijms24010525)
Supplement: Supplementary file 1 [file ijms-24-00525-s001.zip › ijms-2079409-supplementary.pdf]

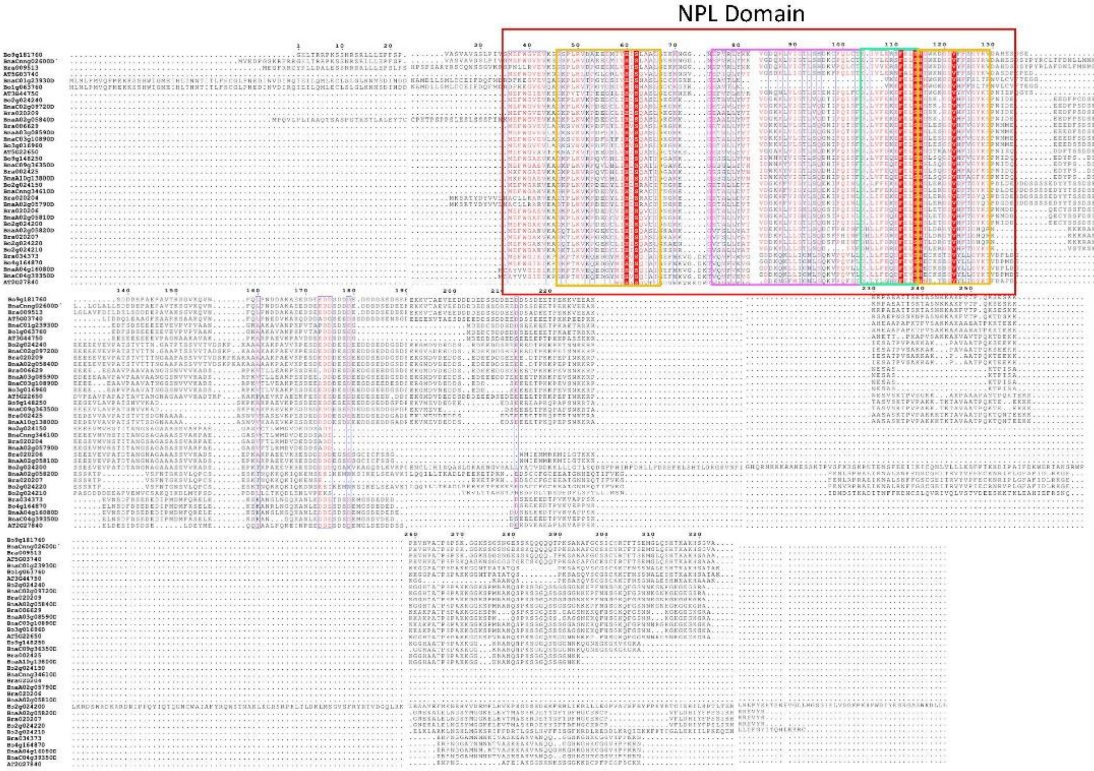

Figure S1. Sequence alignment of HDT proteins.

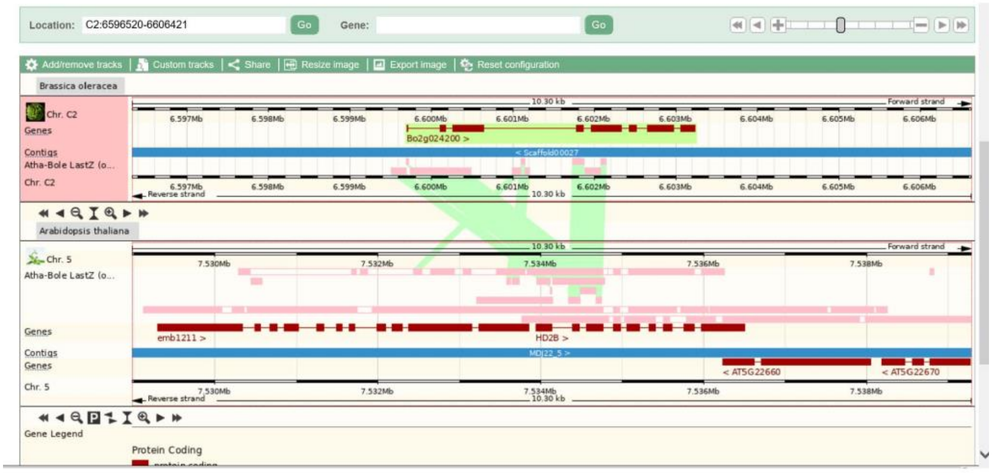

Figure S2. Comparison of gene structure between *ATHDT2* and *Bo2g024200.*

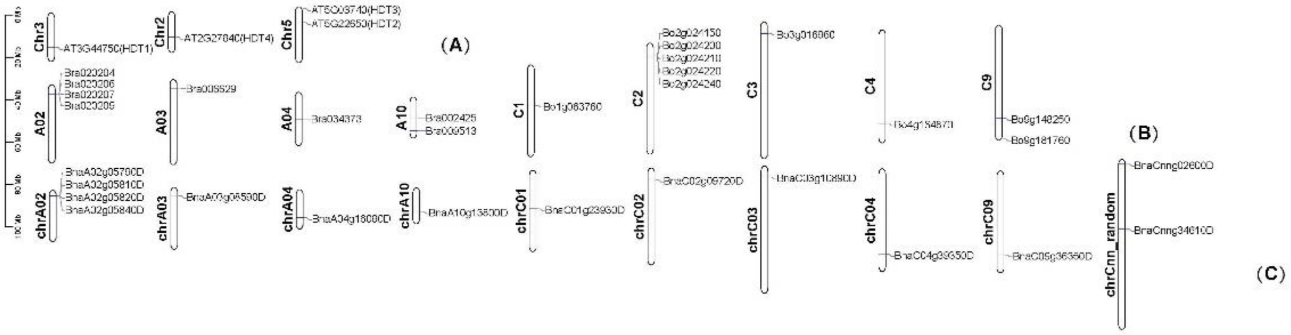

Figure S3. Chromosomal localizations of *HDT* genes in *Arabidopsis* (A), *B. rapa*,(Bra) and *B. oleracea*(Bo) (B), and *B. napus* (C). (A) Chr2,Chr3,Chr5: *Arabidopsis* chromosomes, (B) A02, A03, A04, A10: *B. rapa*, chromosomes; C2, C3, C4, C9: *B. oleracea* chromosomes, (C) ChrA02, chrA03, chrA04, chrA10: *B. napus* An-subgenome chromosomes; ChrC02, chrC03, chrC04, chrC09: *B. napus* Cn-subgenome chromosomes; random means genes were randomly distributed to a specific chromosome. ChrCnn were unanchored scaffolds that could not be mapped to a specific chromosome from the B. napus C-subgenomes.

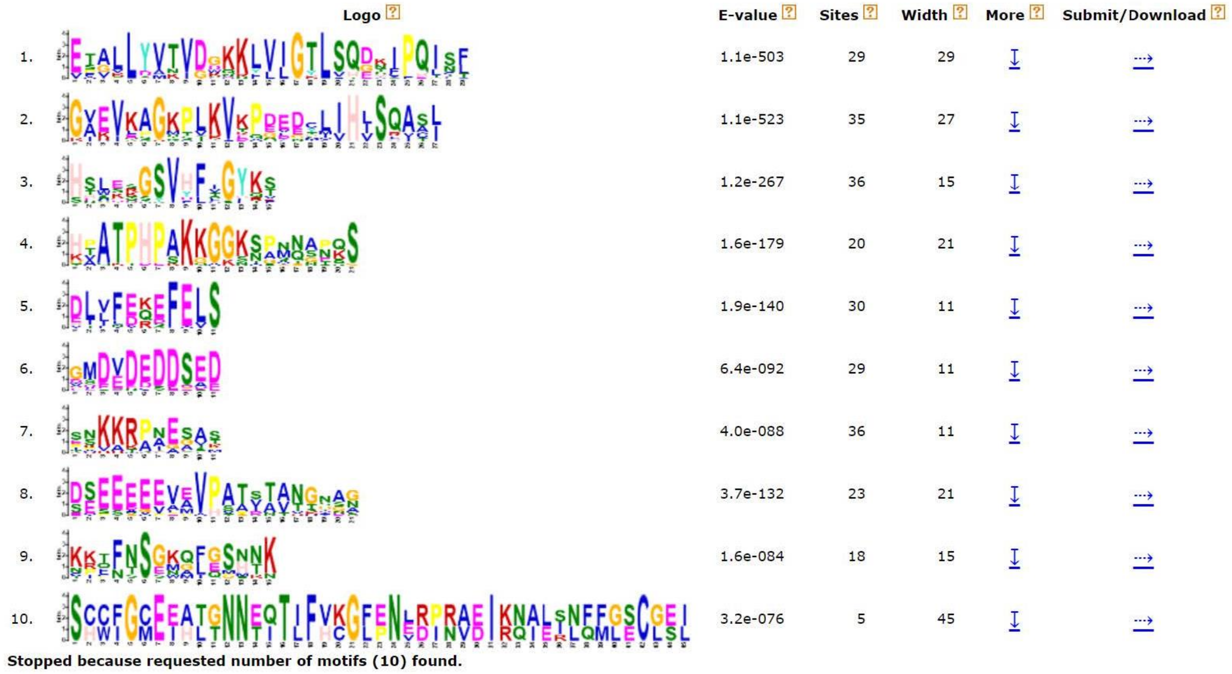

Figure S4. 10 motifs were detected in HDT proteins using MEME.

Table S1. RNA-seq data of expression levels of *AtHDT* , *BraHDT*, *BoHDT* and *BnaHDT* genes in different tissues and organs.

gene_id	root	stem	leaf	bud	sepal	stamen	newpistil	blosomypistil	wiltingpistil	ovule	pericarp	silique
BnaA02g05840D	94.18644	92.0765223	39.00577597	110.228006	23.61232111	340.0437106	7.962097	36.81925	3.398101	130.345	170.4775	329.03
BnaC02g09720D	61.91186	63.9230658	27.44350117	83.2342217	13.94473728	228.8799195	6.064687	10.24578	1.260797	73.90664	95.16839	182.4752
BnaA03g08590D	8.434458	43.0048952	10.36822358	23.2457629	1.464976139	60.94503219	0.661402	0.644645	0	16.24339	17.81137	44.6983
BnaC03g10890D	8.125341	18.3789342	3.609988776	9.34433637	0.627846917	26.69425264	0.955359	1.002781	0.171875	10.85459	12.7343	13.6304
BnaA10g13800D	11.6763	7.91607987	9.241904515	29.2885017	7.716286536	50.18456797	1.161241	4.75364	1.176884	24.08565	22.70864	43.07622
BnaC09g36350D	11.58095	5.07421341	5.415465653	12.3152015	3.688500406	28.31526172	0.932552	1.060412	0.908764	9.850269	13.81821	15.3581
BnaA02g05810D	0.065881	0.2701417	0.563621126	0	0.20815105	0.857767919	0	0.10686	0	1.033651	0.248342	0.560968
BnaA02g05790D	0.069646	0.57115674	0.066203116	0.5807824	0	0.129540461	0	0	0	24.76825	0.7876	2.890987
BnaCnng34610D	0.299092	1.07310891	1.208308413	0.15588485	0	0.278154364	0	0	0	28.54695	0.845583	2.546724
BnaA02g05820D	2.586523	2.81216644	3.352730172	8.53782807	4.828970499	11.00677391	7.761022	3.686843	7.702872	7.446794	11.52277	10.51138
BnaA04g16080D	0.551494	1.1306836	0.996042362	0.40240862	0	4.10309153	0	0.089453	0	1.057557	0.623666	1.056574
BnaC04g39350D	1.523502	2.42939934	1.716377092	0.94108259	0.08913876	7.451575139	0	0.274571	0	2.852643	1.276204	2.522407
BnaC01g23930D	7.840459	7.99924174	6.711139088	15.8420351	1.81973517	38.082131	1.422284	1.988965	0.361566	11.66009	14.28726	23.01806
BnaCnng02600D	31.23818	21.6983567	12.72138315	31.8505053	6.687654181	60.57921567	5.624699	9.192358	2.923319	17.02163	24.70903	40.37062

gene	Root	Leaf	Stem	Sepals	Stamens	Carpels	Flower	Siliques Stage 3	Siliques Stage 4	Siliques Stage 5	Siliques Stage 6
AT3G44750	475.5	130.72	36.77	110.33	76.1	411.3	404.58	106.8	140.05	98.07	106.67
AT5G22650	671.3	324.28	173.33	303.27	236.38	856.32	772.63	368.2	383.08	344.88	384.83
AT5G03740	224.73	115.72	80.03	108.03	155.27	444.3	450.25	225.18	262.1	216.55	260.08
AT2G27840	363.78	146.38	63.2	101.08	58.52	197.95	289	111.02	103.9	94.03	69.4

tracking_id	Root	Stem	Leaf	Bud	Flower	Silique	Callus
Bo1g063760	2.10526	0.603312	8.05516	2.86231	1.3393	2.52561	5.96444
Bo2g024150	0	0.449708	0	0	0.147898	29.2354	0.164663
Bo2g024200	0.068238	0.273772	1.82764	1.91411	1.55314	1.41574	0.150364
Bo2g024210	#N/A	#N/A	#N/A	#N/A	#N/A	#N/A	#N/A
Bo2g024220	6.38207	2.57612	12.5072	8.88869	10.8752	10.2268	7.88907
Bo2g024240	204.962	62.2215	414.987	203.265	33.7984	116.659	273.256
Bo3g016960	29.5496	11.9083	29.0509	22.2023	2.08873	21.1134	19.6697
Bo4g164870	5.72536	1.689	4.12038	6.18558	0.888751	6.37722	11.2555
Bo9g148250	#N/A	#N/A	#N/A	#N/A	#N/A	#N/A	#N/A
Bo9g181760	#N/A	#N/A	#N/A	#N/A	#N/A	#N/A	#N/A
Bra002425	32.2377	40.7451	44.4231	36.9564	21.1477	34.4548	15.5953
Bra006629	40.7167	32.784	84.4606	81.825	31.7366	54.1575	14.612
Bra009513	67.1451	68.7888	46.2489	51.2764	86.3331	85.3799	63.0488
Bra020204	0.293098	0.371959	0.1719345	0.147938	0	32.6815	0
Bra020206	0	0	0	0	0	0.334215	0
Bra020207	0.061075	0	0.0768449	0.0924615	0.183224	1.1041	0.097938
Bra020209	268.5685	476.247	436.6785	467.123	147.943	219.433	168.179
Bra034373	19.3331	16.5746	28.6557	30.5637	8.90675	21.0393	5.71304

Table S2: RNA-seq data of expression levels of *BnaHDT* genes under different abiotic stresses and plant hormones treatments.

gene_id	Leaf_3h	Leaf_6h	Leaf_12h	Cold_3h	Cold_6h	Cold_12h	Hot_3h	Hot_6h	Hot_12h	ABA_3h	ABA_6h	ABA_12h	MeJA_3h	MeJA_6h	MeJA_12h	ET_3h	ET_6h	ET_12h	SA_3h	SA_6h	SA_12h	Root_3h	Root_6h	Root_12h	NaCl_3h	NaCl_6h	NaCl_12h	PEG_3h	PEG_6h	PEG_12h
BnaA02g05840D	50.87044	41.10006	21.66875	10.81015	42.88617	116.9072	40.10827	42.54006	77.3036	38.45993	75.6701	35.09034	48.77822	33.39218	23.45707	74.54782	78.42734	31.02167	42.26686	71.16916	88.32144	92.20299	111.4907	108.6528	67.14072	87.01388	101.9878	109.9265	90.67615	82.03005
BnaC02g09720D	46.73608	41.90791	21.91918	10.16653	16.64047	35.21568	45.59939	59.08086	110.5696	37.96521	60.88262	35.0065	44.44666	33.76137	22.98082	67.92625	67.66269	28.0346	40.87563	62.58869	80.64227	86.57455	106.7216	100.2794	70.59853	93.04877	100.3378	110.5533	96.52524	83.35391
BnaA03g08590D	10.16408	10.19313	3.96233	1.712927	3.025781	3.956875	3.462471	5.616308	4.474285	8.655615	27.38971	3.756039	10.17299	7.72765	3.674061	22.05023	21.06246	4.525841	8.98256	18.27825	22.31134	16.30574	24.52471	24.3978	13.57446	16.68885	24.72666	22.62563	16.89152	15.47884
BnaC03g10890D	2.04359	2.923506	1.087818	0.369194	0.424342	0.99768	0.998239	1.90067	0.982864	1.931876	7.43197	2.208834	3.132322	2.260555	1.103264	4.626858	6.209879	0.99912	1.973581	3.804921	5.682978	5.800381	7.122407	6.067051	3.930444	5.060884	7.637022	7.45459	7.176775	5.023127
BnaA10g13800D	8.148011	11.02681	6.252069	2.551356	3.524051	6.4448	6.991981	7.767372	15.85335	8.711263	17.2208	12.98802	8.913458	9.200378	7.598661	12.78974	19.78068	9.370203	9.13522	12.18477	12.71405	16.54633	18.22405	19.62329	14.59682	15.60389	17.77826	16.495	13.78783	16.11785
BnaC09g36350D	8.632078	6.8144	5.312843	2.515065	4.616369	8.593643	2.958692	3.014731	6.076922	6.154447	10.54439	8.378403	8.16834	5.704766	5.151819	8.516279	11.60037	7.130414	8.215146	11.43572	11.73678	9.993776	14.72228	14.19527	12.91512	13.30416	16.22765	12.04426	10.48805	12.30858
BnaA02g05810D	0.322893	0.617763	0.186553	0.12053	0.202678	0.372868	0.096081	0.43372	0.363357	0.076889	0.611812	0.408393	0.41103	0.27952	0.14146	0.623916	0.927312	0.346995	0.475243	0.278849	0.534174	0	0.031997	0.043298	0.09175	0.140627	0.058931	0	0.053007	0.030604
BnaA02g05790D	0	0	0	0	0	0	0	0	0	0	0	0	0	0	0	0	0	0	0	0	0	0.029591	0.038589	0	0	0.05876	0.140805	0.147021	0.104112	0
BnaCnng34610D	0.116384	0.027296	0.149662	0.189358	0.255947	0.192907	0.049118	0.18161	0	0.094765	0.30387	0.090347	0.032727	0	0	0.030885	0.018445	0.11255	0.11255	0.199409	0.111491	0.139998	0.023718	0.09478	0	0.129717	0.558105	0.124225	0.04615	0.196429

Bna	0.	0.	1.	0.	0.	0.6	1.	1.	2.	0.	1.	1.2	0.8	0.9	1.4	1.	1.	1.	1.	1.	2.	1.	1.	2.1	2.	1.	1.6	1.	1.	2.
A02	99	77	19	76	34	80	44	62	37	91	17	24	69	78	13	11	23	70	73	91	07	67	93	07	37	91	02	92	54	44
g058	75	43	87	70	63	01	67	58	37	66	03	41	98	17	27	64	62	49	87	04	01	24	20	16	18	43	52	65	07	93
20D	31	68	37	2	6	2	05	17	4	06	95	3	8	9	1	38	92	49	48	46	12	19	93	8	62	03	4	25	23	65
Bna	0.	0.	0.	0.	0.	0.6	0.	0.	0.	0.	2.	0.4	0.7	0.6	0.3	1.	1.	0.	0.	1.	2.	1.	3.	2.9	1.	1.	3.0	3.	2.	2.
A04	98	96	30	20	15	79	21	28	36	61	79	89	18	00	41	98	87	35	75	75	29	29	10	15	44	84	44	62	59	90
g160	37	05	22	10	96	88	48	07	01	20	83	07	21	47	90	12	15	46	92	53	13	07	47	71	80	01	14	90	95	07
80D	3	79	36	47	85	4	01	84	54	06	84	6	4	1	6	73	81	74	23	57	58	83	95	3	9	37	5	04	81	46
Bna	3.	2.	1.	0.	1.	2.4	0.	0.	0.	1.	8.	1.8	2.5	1.4	1.1	6.	7.	0.	2.	4.	6.	9.	13		7.	9.	12.	13	8.	9.
C04	22	82	09	85	53	45	51	79	72	94	41	36	98	91	37	41	96	93	40	38	01	23	.2	15.	04	41	75	.0	93	08
g393	45	62	08	39	99	71	94	72	61	72	05	13	82	57	04	74	61	73	37	87	88	87	45	27	82	23	66	24	55	69
50D	7	45	04	9	82	4	89	18	14	95	45	9	2	5	3	8	64	56	59	14	5	12	3	64	38	83	6	02	06	16
Bna	2.	4.	3.	1.	1.	2.1	2.	3.	3.	3.	9.		2.6	2.2	2.2	6.	8.	3.	2.	3.	4.	7.	8.		5.	5.	8.0	7.	7.	7.
C01	76	63	03	19	67	04	38	39	52	21	98	3.9	15	63	67	59	57	47	97	50	06	35	08	7.3	02	01	20	76	99	41
g239	53	39	79	83	16	30	66	59	98	90	74	55	23	96	85	63	41	80	82	18	47	17	69	83	76	17	97	60	44	59
30D	52	29	07	34	22	1	46	29	43	28	74	99	8	4	3	68	98	63	66	09	09	98	44	65	82	34	1	82	53	5
Bna	11	10	11	6.	8.	11.	18	21	49	8.	15	9.9	10.	10.	9.8	12	12	12	13	13	14	35	43	39.	38	40	36.	39	36	41
Cnn	.2	.6	.8	18	10	37	.5	.5	.8	96	.3	05	68	67	91	.5	.8	.5	.2	.7	.3	.9	.9	99	.7	.1	84	.0	.0	.0
g026	50	19	08	72	74	04	56	32	86	27	88	72	46	48	15	10	35	97	06	54	37	47	23	09	63	15	96	93	27	48
00D	9	47	06	94	73	2	44	78	96	08	73	7	8	8	7	26	63	44	1	27	87	52	07	9	36	08	5	03	11	6

Table S3. RNA-seq data of expression levels of *BnaHDT* genes under different low-temperature.

gene_id	HX17_MA	HX17_CA	HX17_FA	HX17_MB	HX17_CB	HX17_FB	HX58_MA	HX58_CA	HX58_FA	HX58_MB	HX58_CB	HX58_FB
BnaA02g05840D	11.67546	24.79769	8.925661	27.69888	48.62542	86.74409	4.498591	13.08269	12.25774	10.23339	95.06071	126.0484
BnaC02g09720D	25.63816	41.75628	16.0856	62.25208	120.0346	134.2699	8.203703	16.7466	14.86383	16.61071	157.5789	138.8114
BnaA03g08590D	2.734362	4.424089	2.022714	8.152468	10.34699	8.970388	0.287605	1.196378	1.419705	1.732016	16.97928	15.52134
BnaC03g10890D	0.108457	0.605434	0.010534	0.584349	3.094805	2.649393	0	0	0.061007	0.093199	2.580671	4.197495
BnaA10g13800D	7.191454	10.12443	4.257001	9.483133	29.20542	27.08059	0.303878	0.22119	1.0235	0.924607	9.994659	9.749105
BnaC09g36350D	6.354079	11.84805	3.47475	9.588258	17.1129	18.50291	3.906548	5.284188	5.548681	5.912688	33.18358	22.96896
BnaA02g05810D	0.017889	0.070231	0	0.085415	0.068321	0.015811	0	0	0	0.014942	0.111754	0.013485
BnaA02g05790D	0	0	0	0	0	0.088032	0.013094	0	0.014063	0.014602	0	0.014053
BnaCnng34610D	1.395851	0.595493	0.69993	3.799195	1.438766	4.145075	3.987993	2.225652	3.036752	5.419788	0.181318	0.47019
BnaA02g05820D	2.180877	2.086215	1.451145	2.996337	4.591788	3.165979	2.597661	3.321969	1.273094	3.344828	4.989705	3.617936
BnaA04g16080D	0.833191	1.262734	0.345504	1.249651	4.272226	4.023432	0.083747	0.439651	0.380718	0.666794	4.268026	6.821455
BnaC04g39350D	8.829801	13.73429	5.246243	7.507761	24.63128	22.77567	1.716244	4.482435	2.286339	3.387485	57.89643	39.61481
BnaC01g23930D	3.602764	7.35241	3.534618	7.857723	14.42529	12.33155	1.313597	2.866638	2.292368	2.624625	44.51624	43.20122
BnaCnng02600D	0	0	0	0	0	0	0	0	0	0	0	0

Table S4. RNA-seq data of expression levels of *BnaHDT* genes infected with S. sclerotiorum.

gene_id	**Westar Control**	**Westar 24hpi**	**ZY821 Control**	**ZY821 24hpi**
BnaA02g05840D	0.356186333	**0.1335198**	**1.88366**	**0.175538667**
BnaC02g09720D	5.86514	23.81336667	1.685676	4.020363333
BnaA03g08590D	1.912766	8.579573333	2.2625	42.1359
BnaC03g10890D	0.280070367	5.966473333	1.35687	32.27626667
BnaA10g13800D	5.559086667	17.3852	7.6069	45.82286667
BnaC09g36350D	6.08331	14.34291	4.874573333	31.01666667
BnaA02g05810D	0.806768	1.386803333	0.327372667	0
BnaA02g05790D	0	0.034526333	0	0
BnaCnng34610D	0.459594333	0.377515	0	0
BnaA02g05820D	4.041793333	5.455313333	0.5788571	0.132761667
BnaA04g16080D	0.386425667	3.363276667	0.516782333	15.30636667
BnaC04g39350D	#N/A	#N/A	#N/A	#N/A
BnaC01g23930D	1.105142333	4.825896667	1.69495	10.17909
BnaCnng02600D	8.529536667	13.13366667	8.825866667	28.03213333
